# MCRIP1 promotes the expression of lung-surfactant proteins in mice by disrupting CtBP-mediated epigenetic gene silencing

**DOI:** 10.1038/s42003-019-0478-3

**Published:** 2019-06-20

**Authors:** Jane S. Weng, Takanori Nakamura, Hisashi Moriizumi, Hiroshi Takano, Ryoji Yao, Mutsuhiro Takekawa

**Affiliations:** 1 Division of Cell Signaling and Molecular Medicine, Institute of Medical Science, The University of Tokyo, Minato-ku, Tokyo 108-8639 Japan; 2 Department of Computational Biology and Medical Sciences, Graduate School of Frontier Sciences, The University of Tokyo, Kashiwa, Chiba 277-8583 Japan; 3 Division of Cell Biology, Cancer Institute, Japanese Foundation for Cancer Research, Koto-ku, Tokyo 135-8550 Japan

**Keywords:** Development, Gene silencing, Differentiation

## Abstract

Proper regulation of epigenetic states of chromatin is crucial to achieve tissue-specific gene expression during embryogenesis. The lung-specific gene products, surfactant proteins B (SP-B) and C (SP-C), are synthesized in alveolar epithelial cells and prevent alveolar collapse. Epigenetic regulation of these surfactant proteins, however, remains unknown. Here we report that MCRIP1, a regulator of the CtBP transcriptional co-repressor, promotes the expression of SP-B and SP-C by preventing CtBP-mediated epigenetic gene silencing. Homozygous deficiency of *Mcrip1* in mice causes fatal respiratory distress due to abnormal transcriptional repression of these surfactant proteins. We found that MCRIP1 interferes with interactions of CtBP with the lung-enriched transcriptional repressors, Foxp1 and Foxp2, thereby preventing the recruitment of the CtBP co-repressor complex to the SP-B and SP-C promoters and maintaining them in an active chromatin state. Our findings reveal a molecular mechanism by which cells prevent inadvertent gene silencing to ensure tissue-specific gene expression during organogenesis.

## Abstract (web summary)

Jane Weng et al. show that a MCRIP1, a regulator of the CtBP transcriptional co-repressor, promotes the expression of lung-specific surfactant proteins SP-B and SP-C, which prevent alveolar collapse. They show that MCRIP1 deficiency in mice causes fatal neonatal respiratory disease by inducing epigenetic silencing of SP-B/C.

## Introduction

Multicellular organisms can create functionally unique cell types or tissues from a single stem cells, even though all cells within an organism share the same genome sequence. This fundamental feature of multicellular life is accomplished through the capacity of individual cell types to generate specific gene expression patterns during embryonic development^1,2^. In mammals, gene expression is rigorously controlled by the coordinated interplay of multiple classes of transcriptional regulatory proteins (e.g., transcription factors, repressors, and coregulators) and the epigenetic status of chromatin (e.g., post-translational histone modifications and genomic DNA methylation)^3,4^. To positively or negatively regulate specific sets of genes, transcriptional factors, or repressors directly bind to unique DNA sequences in the promoter region of their target genes. When bound to DNA, they in turn recruit coregulatory proteins (i.e., coactivators or co-repressors) that form a complex with histone modification enzymes to modulate chromatin structure and transcription. Accumulating evidence has shown that epigenetic modification of chromatin is essential for cells to acquire cell type-specific gene expression and functions^5^. Thus, epigenetic regulation of gene expression has a critical role in a wide range of biological processes including cell differentiation, organogenesis, and morphogenesis. However, molecular mechanisms underlying epigenetic regulation of gene expression during embryonic development are not well defined.

In vertebrates, the C-terminal-binding protein (CtBP) family proteins, CtBP1 and CtBP2, serve as core components of a nuclear transcriptional co-repressor complex that contains chromatin-modifying enzymes such as histone deacetylases (HDAC1/2/4/6/7) and histone methyltransferases (G9a and GLP)^6–8^. Although CtBP proteins do not themselves directly bind to DNA, they can be recruited to promoter regions by interacting with DNA-binding transcriptional repressors that possess a consensus CtBP-binding motif (PxDLS or similar sequences)^9^. Thus, CtBP proteins form a bridge between these transcriptional repressors and chromatin-modifying enzymes, thereby altering the pattern of histone modifications to generate a repressive chromatin structure in the promoters of target genes^6^. Previous reports have demonstrated that CtBP proteins have important roles in a diverse spectrum of biological processes, including cell differentiation, metabolism, and DNA-damage responses, by cooperating with various CtBP-binding transcription repressors such as ZEB1/2, Evi1, and PRDM16^7,10^. Furthermore, a recent genome-wide analysis of CtBP-target genes indicated potential roles for CtBP proteins in embryogenesis^11^. Indeed, in vivo studies using CtBP1 and CtBP2-null mouse models revealed that these two CtBP paralogues had overlapping and distinctive roles during embryonic development. CtBP1-null mice appeared small but viable, whereas CtBP2-null mice were embryonic lethal at embryonic day (E) 10.5^12^. However, the precise role and regulation of CtBP-mediated gene silencing during embryogenesis remain obscure.

Forkhead box P (Foxp) proteins are a family of transcriptional repressors that share a conserved forkhead or winged-helix DNA-binding domain^13^. They bind to regulatory elements of a wide range of genes to spatially and temporally control their expression. In mammals, there are four members of the Foxp family of proteins; Foxp1-4. Although Foxp3 expression is restricted to the T-cell lineage^14^, Foxp1/2/4 are highly expressed in the central nervous system as well as in multiple endodermal cell lineages including the lung^15,16^. In particular, previous studies have shown that Foxp1/2/4 are expressed at high levels in the developing airway epithelium, and play cooperative roles in the regulation of lung endoderm differentiation^16–18^. Foxp2-null mice exhibit defects in postnatal alveolarization. Foxp2^−/−^; Foxp1^+/–^ compound mutant mice die at birth owing to respiratory failure caused by increased severity of airway morphogenesis and differentiation defects^18^. Furthermore, complete loss of Foxp1/2/4 in early lung endoderm results in a dramatic inhibition of normal lung branching and development^19^. Thus, the Foxp family proteins are essential for lung differentiation. In contrast to these in vivo observations, however, previous studies have shown that Foxp1 and Foxp2 proteins interact with transcriptional co-repressors including CtBP, and synergistically repress, rather than promote, the transcription of genes that are essential for lung development and function^16,20–23^. Indeed, Foxp1/2 contain the CtBP-binding motif (PxNLV) and have been shown to cooperate with CtBP to downregulate the expression of lung-specific genes such as SP-C, CCSP (also known as CC10), and Nkx2.1^15,16^. These apparently paradoxical findings have, however, remained unexplained.

We have recently identified the MAPK-regulated co-repressor interacting protein 1 (MCRIP1) as a regulator of the co-repressor function of CtBP^24^. MCRIP1 is a relatively small protein of 97 amino acids that possesses no other known structural domain apart from the C-terminal CtBP-binding motif (PxDLS). We demonstrated that MCRIP1 bound to CtBP, thereby competitively inhibiting the interaction of CtBP with ZEB1 in human epithelial MCF-10A cells and several other cell types. Thus, MCRIP1 prevents CtBP-mediated transcriptional repression of the ZEB1-target genes such as E-cadherin. As CtBP is involved in various biological phenomena, MCRIP1-mediated regulation of CtBP might also impact on a broad range of biological processes. However, the role of MCRIP1 in embryonic development is totally unknown.

In this report, to uncover the physiological function of MCRIP1 in vivo, we generated *Mcrip1*-knockout (KO) mice. The *Mcrip1-*KO mice died shortly after birth owing to respiratory failure that resembles human infant respiratory distress syndrome (RDS). Analysis of the neonatal mice revealed that the levels of the pulmonary surfactant proteins, SP-B and SP-C, which are essential for proper inflation of alveoli, were decreased in *Mcrip1-*KO lungs. We show that MCRIP1 is highly expressed in lung epithelium during alveolar sac formation, and promotes the expression of the surfactant proteins by preventing Foxp1/2 and CtBP-mediated epigenetic gene silencing. Thus, the repression of the surfactant proteins by Foxp1/2 most likely takes place only when MCRIP1 is absent in vivo. Our results demonstrate the critical role of MCRIP1 in lung development and function, and delineate a molecular mechanism that prevents cells from inappropriate gene silencing to generate tissue-specific gene expression during organogenesis.

## Results

### Mcrip1-KO mice die at birth owing to respiratory failure

**Fig. 1:**
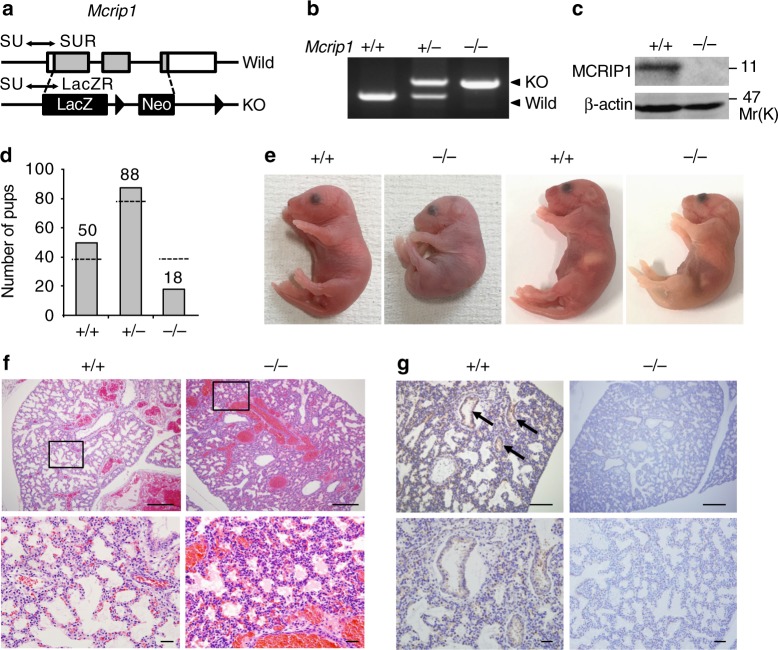
*Mcrip1*-KO mice die at the neonatal stage due to respiratory failure. **a** The targeting strategy for *Mcrip1*-KO mouse generation. Positions of the PCR primers (SU, SUR, and LacZR; for details see methods) that were used for genotyping are indicated. **b** PCR analysis of genomic DNA isolated from *Mcrip1* wild-type (+/+), heterozygous (+/−), or homozygous (−/−) KO mice. **c** Immunoblot analysis of MCRIP1 expression in MEFs isolated from *Mcrip1*^+/+^ or *Mcrip1*^−/−^ mice. β-actin, loading control. **d** Genotype distribution of mice from a pool of 156 recorded pups that were still alive at P7. The bar graph indicates the observed number of pups; the dashed line indicates the expected number of pups according to Mendel’s law. **e** Gross morphology of *Mcrip1*^+/+^ and *Mcrip1*^−/−^ newborn mice. **f** Hematoxylin and eosin staining of lungs from *Mcrip1*^+/+^ or *Mcrip1*^−/−^ mice at P0. The areas in the small squares in the upper panels were enlarged and are shown below. **g** Immunohistochemical analysis of MCRIP1 expression in *Mcrip1*^+/+^ and *Mcrip1*^−/−^ lungs. Lung tissues isolated from the neonates were sectioned and stained with an anti-MCRIP1 antibody. Arrows indicate the MCRIP1-expressing epithelial layer in *Mcrip1*^+/+^ lungs. **f**, **g** Scale bars, 100 μm (upper), 50 μm (lower)

In order to uncover the physiological function of MCRIP1 in vivo, we established *Mcrip1*-KO mice. *Mcrip1* gene-targeted embryonic stem cells were obtained from the Knockout Mouse Project, and were used to create chimeras and to further develop heterozygous mice. The mice were then back-crossed with C57BL/6 mice for >10 generations. In the KO mice, the *Mcrip1* gene on mouse chromosome 11 was substituted with the bacterial β-galactosidase (*LacZ*) reporter gene and a neomycin selection cassette (Fig. 1a). Disruption of the *Mcrip1* gene in heterozygous (+/−) and homozygous (−/−) mutant mice was confirmed by PCR genotyping using genomic DNA and a pair of primers designed to detect both wild-type and mutant alleles (Fig. 1b). Mouse embryonic fibroblasts (MEFs) were isolated from embryonic day 14.5 (E14.5) embryos and MCRIP1 expression was examined at the protein level by immunoblotting using an anti-MCRIP1 antibody. As anticipated, no MCRIP1 protein expression was detected in *Mcrip1*^−/−^ MEFs (Fig. 1c).

Although heterozygous *Mcrip1*^+/−^ mice were alive and as fertile as their wild-type littermates, most of the *Mcrip1*^−/−^ newborn pups died shortly after birth. An analysis of 156 1-week-old pups resulting from heterozygous mating revealed that the distributions of the *Mcrip1* mutant genotype did not follow the Mendelian law^25^ (Supplementary Fig. 1a). The number of pups that survived for at least 1 week was greatly decreased in *Mcrip1*^−*/*−^ mice compared with *Mcrip1*^+/+^ and *Mcrip1*^+/−^ mice (*Mcrip1*^+/+^, 50: *Mcrip1*^+/−^, 88: *Mcrip1*^−/−^, 18) (Fig. 1d), indicating that more than half of *Mcrip1-*KO newborn pups died in the neonatal period. The *Mcrip1*^−/−^ pups had significantly reduced body weight compared with that of their wild-type littermates (*p* = 0.0154) (Supplementary Fig. 1b). To elucidate the cause of death, we more closely monitored the neonates every 2 hours after birth. We found that most of the *Mcrip1*^−/−^ newborn pups exhibited gasping breath, tachypnea and cyanosis, and died within 12 hours after birth (Fig. 1e; Supplementary Movie 1), suggesting that the observed lethality resulted from respiratory failure.

To further analyze the phenotype of MCRIP1 deficiency, we next conducted a whole-body histological survey of *Mcrip1*^−*/*−^ mice. In *Mcrip1*^−/−^ neonates, the lungs were relatively deflated and the alveolar sac space was smaller than that of wild-type lungs (Fig. 1f), suggesting that the observed respiratory failure was caused by impaired inflation of alveoli. Immunostaining of wild-type lungs with an anti-MCRIP1 antibody showed that MCRIP1 was highly expressed in the epithelial layer of alveolar sacs (Fig. 1g). In *Mcrip1*^−/−^ mice, there was no sign of alveolar endothelial cell abnormalities or of vascular leakage in the lungs, and tissues other than the lung appeared normal (Fig. 1f; Supplementary Fig. 1c). These findings thus indicate that MCRIP1 expression in pulmonary epithelia is a prerequisite for the robust inflation of alveolar sacs at birth and that genetic depletion of MCRIP1 leads to postnatal lethality caused by impaired lung inflation and the resulting respiratory failure.

### Surfactant proteins are downregulated in *Mcrip1-*KO lungs

**Fig. 2:**
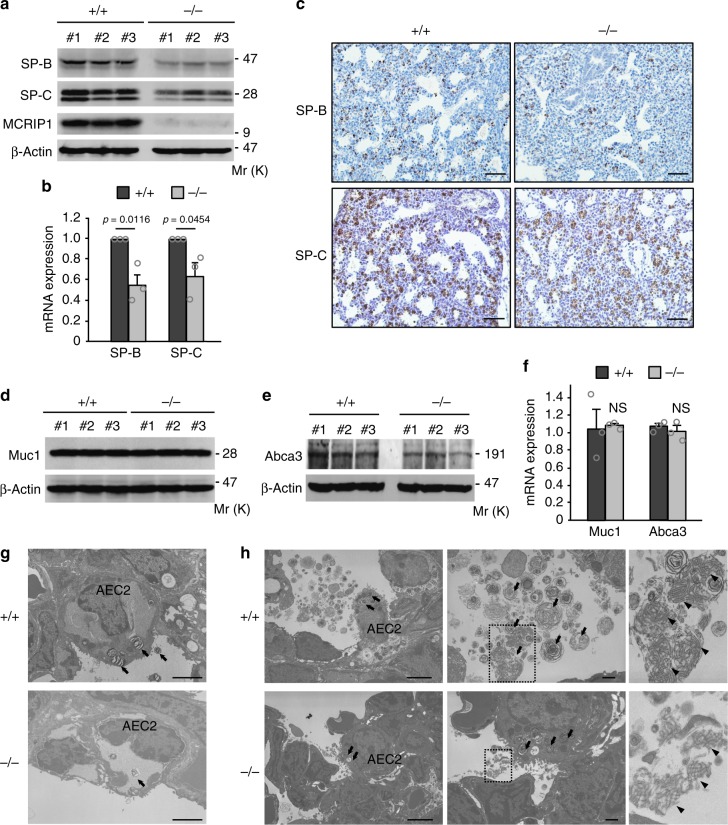
Surfactant proteins are down-regulated in *Mcrip1-*KO mouse lungs. **a**, **b** Immunoblotting **a** and qRT-PCR **b** analyses of SP-B and SP-C expression in whole-lung lysates isolated from *Mcrip1*^+/+^ and *Mcrip1*^−/−^ mice. Data in **b** represent the mean ± SEM. obtained from three mice. **c** Immunohistochemical analyses of SP-B and SP-C expression in the lung tissues of *Mcrip1*^+/+^ and *Mcrip1*^−/−^ neonates. Scale bars, 100 μm. **d**–**f** Immunoblot **d**, **e** and qRT-PCR **f** analyses of the expression of Muc1 and Abca3 in whole-lung lysates isolated from *Mcrip1*^+/+^ and *Mcrip1*^−/−^ mice. Data in **f** represent the mean ± SEM obtained from three mice. NS, not significant. **g**, **h** Ultrastructure of AEC2, lamellar bodies, and tubular myelin in *Mcrip1*^+/+^ and *Mcrip1*^−/−^ lungs at E21 **g** and at P0 **h**. **h** The areas in the small squares in the middle sets of panels were enlarged and are shown on the right. Arrows indicate lamellar bodies and arrowheads indicate tubular myelin. Scale bars, 2 μm in **g**. Scale bars, 2 and 1 μm in left and middle panels of **h**, respectively

Pulmonary surfactant proteins, particularly the hydrophobic surfactant proteins, SP-B and SP-C, prevent alveolar collapse by reducing the surface tension of alveolar fluid and thus they have a pivotal role in alveolar expansion during inhalation^26,27^. Therefore, we next examined if MCRIP1 depletion might affect the expression of these surfactant proteins. For this purpose, we isolated the lungs of newborn mice and examined their extracts for the expression of SP-B and SP-C at both protein and mRNA levels. Western blotting and qRT-PCR analyses of these lung extracts revealed that the expression levels of SP-B and SP-C proteins (Fig. 2a; Supplementary Fig. 2a) and mRNAs (*p* = 0.0116 for SP-B and *p* = 0.0454 for SP-C) (Fig. 2b) were remarkably decreased in *Mcrip1*^−/−^ mice, compared with wild-type mice. Furthermore, immunohistochemical analyses of the lung tissues of *Mcrip1*^−*/*−^ mice showed the relatively weak staining of SP-B and SP-C proteins in alveolar epithelial cells compared with wild-type cells (Fig. 2c). Thus, these data indicated that the respiratory failure observed in *Mcrip1*^−*/*−^ neonates was caused by a deficiency of these surfactant proteins.

Surfactant protein synthesis and secretion occur primarily in type 2 alveolar epithelial cells (AEC2s)^27–29^. In AEC2s, synthesized surfactant proteins are packaged into intracellular storage granules called lamellar bodies, which are then transported to the extracellular space by exocytosis^30^. After secretion onto the alveolar surface, lamellar bodies unfold into a tubular structure, termed tubular myelin, which gives rise to the lipid/protein film at the air-liquid interface to reduce surface tension. As AEC2s are responsible for surfactant protein production, the reduced SP-B and SP-C levels of *Mcrip1*^−/−^ mice may be caused by impaired differentiation of AEC2s in their lungs. Alternatively, it is also possible that MCRIP1 depletion does not affect AEC2 differentiation, but selectively inhibits the synthesis of these surfactant proteins in AEC2s. To distinguish between these two possibilities, we next examined if AEC2 differentiation was altered in *Mcrip1*^−/−^ mouse lungs. For this purpose, we analyzed the expression levels of cell surface associated mucin 1 (Muc1) and of the ATP-binding cassette transporter A3 (Abca3), which are the early and late differentiation markers, respectively, of AEC2s^31^, using immunoblotting (Fig. 2d, e) and qRT-PCR (Fig. 2f). However, no obvious differences in the expression of Muc1 or Abca3 at the protein and mRNA levels were detected between *Mcrip1*^*+/+*^ and *Mcrip1*^−/−^ lungs, indicating that MCRIP1 depletion did not affect AEC2 differentiation. Furthermore, using electron microscopy, we investigated the ultrastructure of AEC2s in *Mcrip1*^*+/+*^ and *Mcrip1*^−/−^ mouse lungs before (E21) (Fig. 2g) and after birth (P0) (Fig. 2h). Although the distribution of AEC2s was similar in the lungs of both mice, the number and the size of the cytoplasmic lamellar bodies in individual AEC2s were markedly decreased in the *Mcrip1*^−/−^ mice versus those in the *Mcrip1*^*+/+*^ mice (Fig. 2g, h; Supplementary Fig. 2b). Accordingly, the tubular myelin formation was also decreased in the alveolar extracellular space of *Mcrip1*^−/−^ mice versus *Mcrip1*^*+/+*^ mice (Fig. 2h). Interestingly, these phenotypes are similar to those observed in SP-B-deficient mice in that fully formed lamellar bodies were not detected in AEC2s and, reflecting the lack of the major surfactant protein, the mice died shortly after birth owing to respiratory distress^32^. Therefore, we concluded that MCRIP1 depletion did not affect the differentiation of AEC2s, but led to insufficient synthesis of the surfactant proteins B and C in AEC2s.

### MCRIP1 is highly expressed in embryonic lung epithelium

**Fig. 3:**
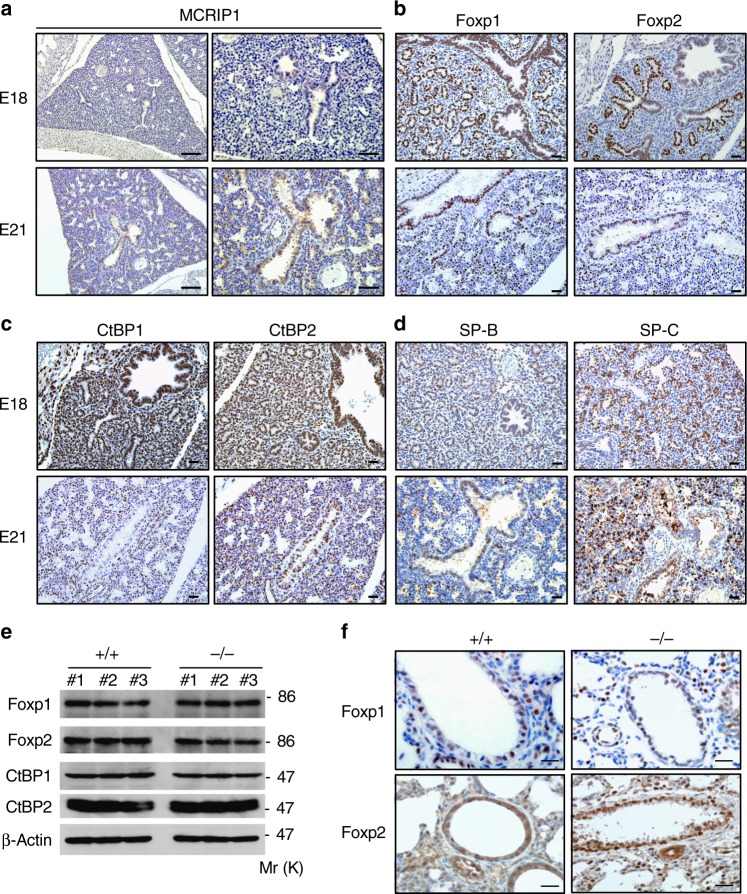
MCRIP1 is expressed in the lung epithelium during lung development. **a**–**d** Immunohistochemical analysis of the expression of MCRIP1 **a**, Foxp1 and Foxp2 **b**, CtBP1 and CtBP2 **c**, and SP-B and SP-C **d** in *Mcrip1*^*+/+*^ lungs at the embryonic stage of E18 and E21. Scale bars, 100 and 50 μm in left and right panels of **a**, respectively. Scale bar, 50 μm in **b**–**d**. **e** Western blot analysis of whole-lung lysates isolated from *Mcrip1*^+/+^ and *Mcrip1*^−/−^ mice. The levels of the indicated proteins were analyzed by immunoblotting with their specific antibodies. β-actin, loading control. **f** Immunohistochemical analysis of Foxp1 and Foxp2 in lung tissues isolated from *Mcrip1*^+/+^ and *Mcrip1*^−/−^ mice. Scale bars, 20 μm

We previously demonstrated that MCRIP1 selectively binds to the transcriptional co-repressor CtBP and inhibits interactions of CtBP with DNA-binding transcriptional repressors harboring the CtBP-binding motif^24^. Although the precise role of CtBP in the regulation of lung development remains unclear, previous studies have shown that the CtBP-binding transcriptional repressors, Foxp1 and Foxp2, are preferentially expressed in alveolar epithelial cells and suppress the expression of SP-C and other lung-related genes^15–18,33^. Therefore, we hypothesized that MCRIP1 might inhibit CtBP and Foxp1/2-mediated transcriptional repression of surfactant proteins in AEC2s. To explore this possibility, we initially monitored the expression patterns of MCRIP1, CtBP1/2, and Foxp1/2 in lung tissues of wild-type mice at critical embryonic stages during lung development, i.e., at E17 (Supplementary Fig. 3a–d), E18, and E21 (Fig. 3a–d), which represent the end of the canalicular stage (when type 1 and type 2 AECs start to emerge), the beginning of the saccular stage (when alveolar sacs form), and the alveolar stage, respectively^34,35^. Immunohistochemistry revealed that MCRIP1 was preferentially expressed in the epithelial layer of the developing lung at all stages tested, indicating that MCRIP1 is constitutively expressed in embryonic lung epithelium during alveolar sac formation (Fig. 3a; Supplementary Fig. 3a). Furthermore, as reported previously^18^, Foxp1 was expressed at high levels in the nucleus of lung epithelial cells and at lower levels in the developing mesenchyme, whereas Foxp2 expression was restricted to distal lung epithelia (Fig. 3b; Supplementary Fig. 3b). CtBP1 and CtBP2 were found to be more widely expressed in the nucleus of various types of cells, but were expressed at particularly high levels in lung epithelial cells (Fig. 3c; Supplementary Fig. 3c). Concomitantly with the development of AECs, SP-B, and SP-C expression was detected in embryonic lungs at E17 and accumulated thereafter (Fig. 3d; Supplementary Fig. 3d). We also found that the depletion of MCRIP1 did not affect the expression levels of these transcriptional regulatory proteins in lungs. Western blot analysis (Fig. 3e) and immunohistochemistry (Fig. 3f) showed that the expression levels of Foxp1/2 and CtBP1/2 in the neonatal (P0) lungs of *Mcrip1*^−/−^ mice were indistinguishable from those in *Mcrip1*^+/+^ control mice. Collectively, these findings demonstrate that, even though Foxp1/2 and CtBP1/2, which cooperate to inhibit surfactant protein expression, are highly expressed in AECs during lung development, AECs can robustly produce these surfactant proteins when MCRIP1 is expressed.

### MCRIP1 competitively inhibits the CtBP-Foxp1/2 interactions

**Fig. 4:**
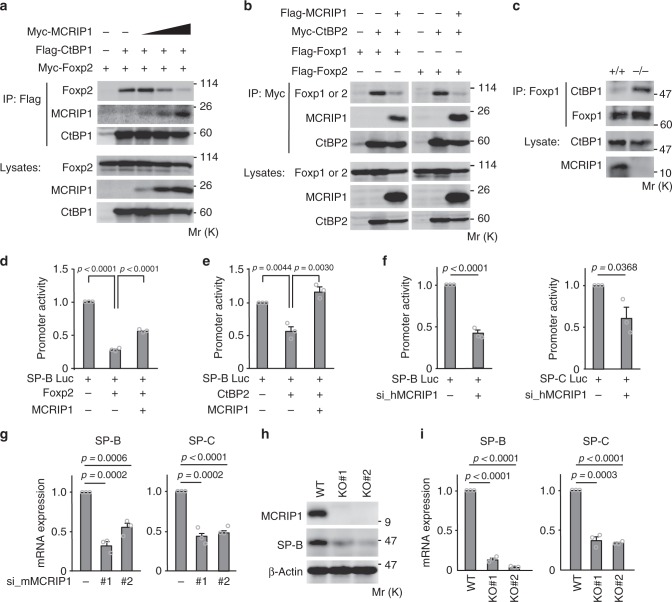
MCRIP1 promotes the expression of SP-B and SP-C by disrupting the CtBP-Foxp interactions. **a**, **b** MCRIP1 inhibits Foxp-CtBP interactions. U2OS cells were transiently transfected as indicated. **a** Flag-CtBP1 was immunoprecipitated, and co-precipitated Myc-MCRIP1 and Myc-Foxp2 were probed with an anti-Myc antibody. Increasing amounts of Myc-MCRIP1 expression vector were used. **b** Myc-CtBP2 was immunoprecipitated, and co-precipitated Flag-MCRIP1, Flag-Foxp1, or Flag-Foxp2 were probed with an anti-Flag antibody. **c** Co-precipitation of endogenous CtBP1 and Foxp1 in lung lysates from *Mcrip1*^+/+^ and *Mcrip1*^−/−^ mice. Endogenous Foxp1 was immunoprecipitated and co-precipitated CtBP1 was detected by immunoblotting. **a**–**c** The levels of protein expression are shown in the lower rows. **d**, **e** MCRIP1 inhibits the repression of the SP-B promoter induced by Foxp2 **d** or CtBP2. **e** A549 cells were transfected with the SP-B-luciferase reporter plasmid (SP-B Luc) and the pMCRIP1 plasmid, together with pFoxp2 **d** or pCtBP2 **e** as indicated, and SP-B promoter activity was analyzed (fold induction). **f** SP-B and SP-C promoter assays. A549 cells were transfected with siRNA targeting human *MCRIP1*, cultured for 48 h, and then re-transfected with SP-B-Luc or SP-C-luciferase reporter plasmid (SP-CLuc). After an additional 48-h incubation, SP-B, or SP-C promoter activities were analyzed. **d**–**f** Data are means ± SEM from three independent experiments performed in triplicate. **g** MCRIP1 siRNA-mediated depletion decreases the mRNA expression of SP-B and SP-C. MLE12 cells were transfected with siRNAs targeting mouse *Mcrip1* (#1 or #2). Total RNA was then extracted and analyzed for endogenous SP-B (left) or SP-C (right) mRNA expression (fold change) using qRT-PCR. **h**, **i** *Mcrip1*-KO MLE12 cells were established using the CRISPR/Cas9 approach with two different guide RNAs (KO#1 or KO#2). **h** The expression levels of endogenous MCRIP1 and SP-B proteins were analyzed by immunoblotting. β-actin, loading control. **i** The expression levels of SP-B and SP-C mRNAs were analyzed using qRT-PCR. **g**, **i** The data represent the mean ± SEM from three independent experiments

Previous studies have shown that both Foxp1 and Foxp2 bind to CtBP through the CtBP-binding motif (PLNLV) and other sites^15,16,36^. To clarify the molecular mechanism by which MCRIP1 promotes surfactant protein expression, we next tested if MCRIP1 affected the interactions between Foxp1/2 and CtBP1/2. Using in vivo co-precipitation experiments, we confirmed that Foxp2 interacted with CtBP1 (Fig. 4a, lane 2). As MCRIP1 also interacts with CtBP through the consensus CtBP-binding sequence^24^, we then tested if MCRIP1 could competitively inhibit the interaction of Foxp1/2 with CtBP. Co-immunoprecipitation experiments demonstrated that coexpression of MCRIP1 with Foxp2 interfered with the Foxp2-CtBP1 interaction in a dose-dependent manner (Fig. 4a). Similarly, MCRIP1 also disrupted the association of CtBP2 with Foxp1/2 (Fig. 4b). Furthermore, the endogenous interaction between Foxp1 and CtBP1 was detected in lung lysates from *Mcrip1*^−/−^ mice, but not in those from *Mcrip1*^+/+^ mice (Fig. 4c), indicating that CtBP1 binds to Foxp1 particularly in the absence of MCRIP1 in mouse lungs. As MCRIP1 did not directly bind to Foxp1/2 (Supplementary Fig. 4a), we concluded that MCRIP1 competed with Foxp1/2 for CtBP binding.

As Foxp1/2 cooperate with CtBP to repress the transcription of target genes^16,20,36^, we next investigated if expression of MCRIP1 could modulate the promoter activities of the SP-B and SP-C genes, by using luciferase reporter assays. For this purpose, we initially utilized lung adenocarcinoma A549 cells, a model cell line for human AEC2s, although they have heterogeneous properties in the expression levels of some AEC markers^37^. Overexpression of Foxp2 significantly repressed SP-B promoter activity in these cells (*p* < 0.0001) (Fig. 4d). Coexpression of MCRIP reversed the Foxp2-mediated repression of SP-B reporter activity (*p* < 0.0001). Similarly, overexpression of CtBP2 resulted in the repression of SP-B promoter activity (*p* = 0.0044) and the coexpression of MCRIP1 abrogated such repression (*p* = 0.0030) (Fig. 4e). Conversely, depletion of endogenous MCRIP1 by an siRNA against human *MCRIP1* resulted in the repression of SP-B and SP-C promoter activities in A549 cells (*p* < 0.0001 for SP-B and *p* = 0.0368 for SP-C) (Fig. 4f, Supplementary Fig. 4b). Consistent with these data, MCRIP1 depletion using two siRNAs targeting different regions of mouse *Mcrip1* indeed suppressed the expression of SP-B and SP-C mRNAs in MLE12 cells, a murine AEC2 cell line^38^ (Fig. 4g; Supplementary Fig. 4c). Virtually identical results were also obtained in the following CRISPR/Cas9-mediated genome-editing experiments. We established two independent *Mcrip1-*knockout MLE12 cell lines (KO#1 and KO#2) using two different guide RNAs (Fig. 4h). In these *Mcrip1*-KO cells, the expression of SP-B protein (Fig. 4h; Supplementary Fig. 4d) and SP-B and SP-C mRNAs (Fig. 4i; Supplementary Fig. 4e) was strongly suppressed, further confirming the importance of MCRIP1 for the robust expression of these surfactant proteins. These combined data indicate that MCRIP1 interfered with the CtBP1/2 and Foxp1/2 interactions and thereby disrupted CtBP-mediated transcriptional repression to promote the expression of SP-B and SP-C.

### MCRIP1 regulates recruitment of CtBP to the SP-B promoter

**Fig. 5:**
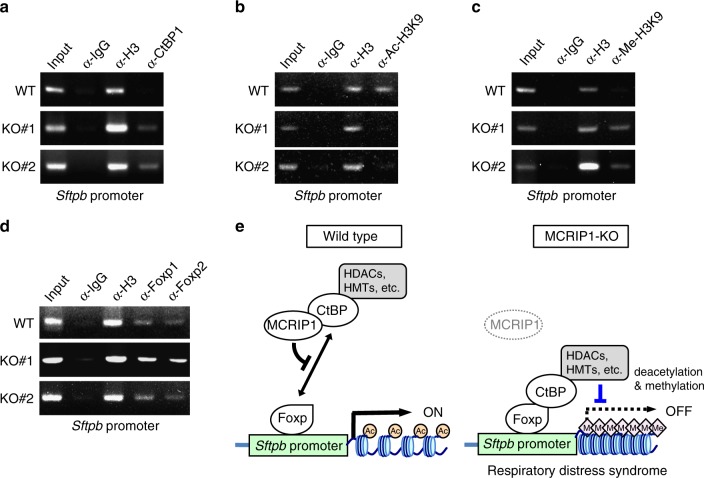
MCRIP1 depletion allows the recruitment of a CtBP co-repressor complex to the SP-B promoter and modulates histone modifications. **a**–**d** Chromatin immunoprecipitation (ChIP) assays were performed using parental (WT) and *Mcrip1*-KO (KO#1 and KO#2) MLE12 cells. Cross-linked chromatin was immunoprecipitated with antibodies specific for CtBP1 **a**, histone H3 acetylated at Lys9 (Ac-H3K9) **b**, histone H3 methylated at Lys9 (Me-H3K9) **c**, or Foxp1, or Foxp2 **d**. Immunoprecipitated DNAs were amplified with PCR primers specific for the SP-B promoter. Rabbit IgG and an anti-Histone H3 antibody were used as negative and positive controls, respectively. The input lanes represent 2% of the total chromatin used for each immunoprecipitation. **e** A schematic model of MCRIP1-dependent epigenetic regulation of SP-B and SP-C expression. In the lung epithelial cells of *Mcrip1*^+/+^ (wild-type) mice, MCRIP1 binds to CtBP and thereby disrupts the interaction of CtBP with DNA-binding transcriptional repressors (e.g., Foxp1/2). As a result, CtBP cannot be recruited to the promoters of target genes (e.g., SP-B and SP-C). In contrast, in *Mcrip1*^−/−^ (MCRIP1-KO) lungs, the absence of MCRIP1 allows CtBP to interact with Foxp1/2 on the promoter regions of SP-B and SP-C. When bound to the promoters, the CtBP co-repressor complex, which includes histone-remodeling enzymes (e.g., HDACs and HMTs), modulates chromatin conformation via deacetylation and methylation of histones, resulting in transcriptional silencing of the surfactant proteins and the induction of respiratory failure that resembles human infant RDS

DNA-binding transcriptional repressors such as Foxp1/2 recruit CtBP and its associated chromatin-modifying enzymes including HDACs and histone methyltransferases (HMTs) to the promoter region of their target genes, where the CtBP complex elicits epigenetic gene silencing by deacetylating and methylating Lys9 of histone H3^6^. To investigate if MCRIP1 affects the recruitment of CtBP to the promoter of the endogenous SP-B gene (*Sftpb*) and the consequent histone modifications, we next performed chromatin immunoprecipitation (ChIP) assays using MLE12 cells and antibodies specific to CtBP1, acetylated histone H3-Lys9 (Ac-H3K9; an active chromatin marker), or methylated histone H3-Lys9 (Me-H3K9; a marker of inactive chromatin). In the wild-type control MLE12 cells, CtBP1 was absent from the *Sftpb* promoter, and therefore the promoter-associated histone H3 was acetylated, but not methylated at Lys9 (Fig. 5a–c, top). In contrast, the ablation of MCRIP1 by CRISPR/Cas9-mediated gene editing led to recruitment of CtBP1 to the promoter region of *Sftpb*, resulting in reduced acetylation of H3K9, but increased H3K9 methylation, probably due to the CtBP-mediated recruitment of HDACs and HMTs to the promoter (Fig. 5a–c, middle and bottom). As might be anticipated, binding of Foxp1 and Foxp2 to the *Sftpb* promoter was not affected by MCRIP1 depletion (Fig. 5d). Overall, these results indicate that MCRIP1 promoted the expression of SP-B and SP-C, which are essential for robust inflation of alveolar sacs at the neonatal stage, by releasing these surfactant genes from CtBP-mediated epigenetic silencing during lung development as summarized in Fig. 5e.

## Discussion

MCRIP1 is a recently identified CtBP-binding protein that modulates CtBP-mediated gene silencing^24^. Although previous studies have shown that CtBP is involved in a broad range of biological processes such as epithelial mesenchymal transition, cellular metabolism and immune responses^7,8,11^, the precise role of the MCRIP1-CtBP axis in the regulation of embryonic development is completely unknown. In this study, to clarify the in vivo function of MCRIP1, we generated *Mcrip1*^−/−^ mice. Analyses of these mice showed that MCRIP1 is a key molecule for the robust expression of the surfactant proteins in the developing lung and is thus critical for the survival of neonatal mice. The majority of *Mcrip1*^−/−^ mice died in the neonatal stage owing to respiratory failure caused by deflation of alveolar sacs. We found that the expression of the surfactant proteins, SP-B and SP-C, which reduces the surface tension of the alveolar air-liquid interface^26,27^, was substantially repressed at both the mRNA and protein levels in AEC2s of *Mcrip1*^−/−^ mice, which results in the collapse of alveoli and small airways.

Insufficient production of surfactant proteins in human newborns leads to a life-threatening condition called infant RDS^27^. Although RDS is usually observed in preterm infants owing to their immature lung development, full-term infants carrying germline mutations in genes that are involved in surfactant production and metabolism can also develop refractory RDS. To date, mutations of the genes encoding SP-B, SP-C, ABCA3, and colony stimulating factor 2 receptor α and β subunits (CSF2RA and CSF2RB) have been identified in some cases of familial RDS^27^, but other causative genes remain to be uncovered. Our results imply that MCRIP1 may be a potential target of mutational inactivation in human hereditary RDS. Further investigation is required to clarify the role of MCRIP1 in the etiology of neonatal respiratory diseases.

Previous reports have shown that the Foxp1 and Foxp2 transcriptional repressors contain the CtBP-binding motif and therefore, in collaboration with CtBP, inhibit the expression of several lung-specific genes including SP-C^15,16^. Furthermore, we found that these repressors also bound to the SP-B promoter, and repressed its activity by recruiting CtBP. However, as shown in this and other studies^16–18^, both Foxp1 and Foxp2 are highly expressed in lung epithelium, including AEC2s that are responsible for surfactant biosynthesis. It is therefore unclear how the surfactant protein expression avoids Foxp1/2 and CtBP-mediated transcriptional repression in AEC2s. Importantly in this respect, we demonstrated here that repression of surfactant protein expression took place only in the absence of MCRIP1. Genetic ablation of endogenous MCRIP1 profoundly inhibited the promoter activity and the expression of SP-B as well as of SP-C in the cultured lung cells and in mouse lung tissues. Conversely, MCRIP1 expression disrupted Foxp1/2 and CtBP-mediated repression of SP-B reporter activity. Based on these findings, we propose the following model for MCRIP1 function during lung development (Fig. 5e). In normal, MCRIP1 wild-type lung epithelial cells, MCRIP1 tightly associates with CtBP via its PxDLS motif and competitively inhibits the interactions between CtBP and Foxp1/2. MCRIP1-mediated inhibition of CtBP-Foxp1/2 interactions prevents the recruitment of CtBP to the promoter regions of SP-B and SP-C, thereby maintaining the associated chromatin in a transcriptionally active state to ensure robust expression of the surfactant proteins. In contrast, without the interference of MCRIP1 in *Mcrip1*^−/−^ lung epithelium, free CtBP can interact with Foxp1/2 bound to the SP-B and SP-C promoters. When recruited to these promoters, the CtBP co-repressor complex, which contains chromatin-remodeling enzymes such as HDACs and HMTs, modulates the chromatin conformation through histone deacetylation and methylation, leading to abnormal transcriptional silencing of SP-B and SP-C and resulting respiratory failure. These findings delineate a molecular mechanism as to how the production of the surfactant proteins, which are essential for lung function and postnatal survival, is protected from unwanted gene silencing during embryonic development. Consistent with this notion, we found that MCRIP1 was expressed at high levels in the lung epithelium as early as E17, when AEC2s start to develop, and that its expression in the lung persisted into postnatal stages. Furthermore, reintroduction of MCRIP1 into *Mcrip1*-KO MLE12 cells did not suffice to restore SP-B expression (Supplementary Fig. 4d, e), which also implies that, once *Mcrip1* has been genetically ablated, the SP-B expression is epigenetically silenced at the level of chromatin remodeling through the orchestrating processes mediated by CtBP and histone modification enzymes. It should be noted that we have previously demonstrated that upon growth factor stimulation MCRIP1 can be selectively phosphorylated by ERK and this phosphorylation inhibits its interaction with CtBP^24^. In this regard, we confirmed that no ERK activation was detected in neonatal lungs (Supplementary Fig. 5), indicating that most MCRIP1 proteins exist in an unphosphorylated state and are thus functional as CtBP inhibitors in lung epithelium.

Several studies have shown that some Foxp family members including Foxp1 can exert their transcriptional repressor function by interacting not only with CtBP but also with other co-repressor complexes such as the nucleosome-remodeling and histone deacetylation chromatin-remodeling complex, and the silencing mediator of retinoic acid and thyroid hormone receptor (SMRT) complex^39,40^. Furthermore, recent genome-wide analyses of Foxp1/2-target genes revealed that Foxp1/2 not only downregulate some sets of genes, but can also upregulate others by acting as transcription factors^41–44^. These findings therefore indicate that Foxp1/2 have the ability to interact with various cofactors (i.e., co-repressors or coactivators) to negatively or positively regulate their target gene expression depending on the cell type and the target promoter. As MCRIP1 specifically binds to CtBP^24^, it must impair the interaction of Foxp1/2 only with CtBP, and thereby it might promote the binding of Foxp1/2 to cofactors other than CtBP. Therefore, MCRIP1 dictates the proper choice of cofactor binding to Foxp1/2 (and probably to other CtBP-binding transcriptional repressors) in developing lung epithelium in order for cells to create gene expression patterns that are necessary for normal lung development and function. As MCRIP1 is preferentially expressed in lung epithelial cells, such MCRIP1-mediated regulation of CtBP occurs in a cell type dependent manner. Thus, our data define a molecular mechanism by which a single transcriptional repressor such as Foxp1/2 can exert various, even opposite, effects on histone modifications and the resulting gene expression depending on the cell type or the tissue-specific context.

Although MCRIP1 is preferentially expressed in lung epithelium, MCRIP1 expression is also detected in other tissues, including in brain, liver, and pancreas^24^. Nevertheless, *Mcrip1*^−/−^ mice showed functional defects only in the lung. This observation might be explained by the presence of MCRIP2 (Gene ID: 68241), a paralog of MCRIP1, in mammals. Although the precise function of MCRIP2 is unknown, it also possesses the consensus CtBP-binding motif, suggesting a redundant role for these two paralogues in the regulation of CtBP. Therefore, MCRIP1 depletion might be partially compensated for by MCRIP2 in other tissues. Indeed, according to the Atlas protein database, the expression level of MCRIP2 in lung is relatively low, compared with the level of MCRIP1 expression. The generation of *Mcrip1*/*2* double knockout mice may deepen our understanding of the distinct and overlapping functions of these two paralogues.

In summary, we demonstrated that MCRIP1 is essential for the robust production of the pulmonary surfactant proteins by regulating CtBP-Foxp1/2 interactions and chromatin remodeling at their target genes, including SP-B and SP-C, in lung epithelium. Thus, MCRIP1 depletion elicits abnormal epigenetic silencing of these surfactant protein genes, resulting in fatal respiratory failure at birth, which resembles human RDS. Our data delineate a molecular mechanism by which MCRIP1 modulates specific transcriptional programs in alveolar epithelial cells, and highlight the importance of epigenetic regulation of gene expression during organogenesis.

## Methods

### *Mcrip1-*knockout mice

*Mcrip1-*knockout ES cells were obtained from the Knockout Mouse Project Repository. *Mcrip1-*knockout mice were constructed according to the targeting strategy outlined in Fig. 1a. Heterozygous *Mcrip1* males were mated with heterozygous *Mcrip1* females to obtain homozygous *Mcrip1-*knockout mice. Genomic DNA was extracted from mouse tail tissues using CellEase Mouse Tail (Biocosm) according to the manufacturer’s protocol and was used for genotyping PCR. The following primers were used for genotyping PCR: *SU* Forward, 5′-TTGGAGAAGCATGCACAGTC-3′; *SUR* Reverse, 5′-CATTCACCTGAGACCCACCT-3′; *LacZ* Reverse, 5′-GTCTGTCCTAGCTTCCTCACTG-3′. All animal experiments were conducted in accordance with the Regulations for Animal Care and Use of The University of Tokyo and the Guidelines for Proper Conduct of Animal Experiments by the Science Council of Japan, and were approved by the Animal Experiment Committee at the Institute of Medical Science, The University of Tokyo (approval number: A17-81).

### Plasmids

Flag-MCRIP1, Myc-MCRIP1, Flag-CtBP1, Myc-CtBP1, Flag-CtBP2 and Myc-CtBP2 expression plasmids were described previously^24^. Flag-Foxp1, Flag-Foxp2, Myc-Foxp1, and Myc-Foxp2 were sub-cloned into the pcDNA3 vector (Invitrogen) using PCR-based methods. Foxp2 mutants were constructed using PCR-based site-directed mutagenesis. SP-B and SP-C promoter constructs were sub-cloned into the pGL4.21 luciferase reporter vector (Promega).

### Media and buffers

Lysis buffer used for co-immunoprecipitation experiments contained 20 mM Tris-HCl (pH7.5), 10% glycerol, 100 mM NaCl, 0.5 mM EDTA, 54 mM β-glycerophosphate, 10 mM NaF, 2 mM sodium vanadate, 1 mM dithiothreitol, 0.5 mM phenylmethylsulphonyl fluoride (PMSF), 10 μg mL^−1^ leupeptin, 10 μg mL^−1^ aprotinin and 0.5% NP-40. The lysis buffer used for mouse tissue lysate extraction contained 50 mM Tris-HCl (pH7.5), 150 mM NaCl, 1 mM EDTA, 54 mM β-glycerophosphate, 10 mM NaF, 2 mM sodium vanadate, 1 mM dithiothreitol, 1 mM PMSF, 10 μg ml^−1^ leupeptin, 10 μg ml^−1^ aprotinin and 1.0% TritonX-100. Sodium dodecyl sulfate polyacrylamide gel electrophoresis (SDS-PAGE)-loading buffer consisted of 65 mM Tris-HCl (pH6.8), 5%(v/v) 2-mercaptoethanol, 3% SDS, 0.1% bromophenol blue and 10% glycerol.

### Cell culture and transient transfection

U2OS cells were cultured in RPMI1640 medium. A549 and MEF cells were maintained in DMEM with 4.5 g L^−1^ d-(+)-glucose. Both RPMI1640 medium and DMEM with 4.5 g L^−1^ d-(+)-glucose medium were supplemented with 10% fetal bovine serum (FBS), l-glutamate, and penicillin–streptomycin. MLE12 cells were maintained in DMEM/F-12 medium supplemented with 2% FBS, 5 μg mL^−1^ insulin, 10 μg mL^−1^ transferrin, 30 nm sodium selenite, 10 nm β-estradiol, and 10 nm hydrocortisone, as instructed by the American Type Culture Collection (ATCC). For transient transfection, pre-seeded U2OS and A549 cells were transfected with their respective expression plasmids using the X-treme Gene 9 DNA Transfection Reagent (Roche).

### Electron microscopy of AEC2s

Neonatal lung tissues were isolated from *Mcrip1*^*+/+*^ and *Mcrip1*^−*/*−^ mice at E21 and were cut into pieces. Cut tissues were fixed with 2.5% glutaraldehyde, 2% formaldehyde, and 0.1 m sodium phosphate (pH7.4) for 2 h at room temperature. After washing in 3% sucrose in 0.1 m sodium phosphate buffer, the samples were post-fixed in 1% osmium tetroxide in 0.1 m phosphate buffer for 2 h on ice. The samples were then dehydrated in a graded series of ethanol and embedded in Epon 812 resin mixture (TAAB Laboratories). Samples were continuously cut with an Ultracut S ultramicrotome (Leica) into serial ultrathin sections. The resultant series of ultrathin sections were placed on a single-slot copper grid. Then, the ultrathin sections were then stained with 2% uranyl acetate in 70% ethanol for 5 min at room temperature, followed by staining in Raynold’s lead for 5 min at room temperature. The stained sections were examined under a transmission electron microscope (Hitachi H-7500).

### Co-immunoprecipitation assay

Cell lysates were harvested in lysis buffer. The appropriate antibody was added into the cell lysates and incubated at 4 °C for 2 h with gentle rotation. Protein G-sepharose beads (GE) were then added and incubated at 4 °C for an additional 1 h. Immunoprecipitants were collected by centrifugation and washed three times with lysis buffer. Proteins were separated by SDS-PAGE and immunoblotted with the indicated antibodies.

### Immunoblotting analyses

Immunoblotting analyses were carried out as described previously^24^. Digital images were captured using the LAS-1000 Plus (Fujifilm). The following primary antibodies were used: mouse anti-Flag M2 (Sigma), anti-Myc 9E10 (Santa Cruz), and anti-β-actin 2F3 (Wako); rabbit anti-Myc and anti-SP-B (Santa Cruz), anti-CtBP1, anti-CtBP2, anti-SP-C, anti-Muc1 and anti-Abca3 (Abcam), anti-Foxp1 (CST), anti-Foxp2 (Sigma), anti-phospho-ERK1/2 (CST), and anti-ERK2 (Santa Cruz). An affinity-purified anti-MCRIP1 antibody was made in-house as described previously^24^. All antibodies were used at a dilution of 1:1000 for western blotting. Full size western blot images are shown in Supplementary Fig. 6.

### Immunohistochemistry

Tissues from neonatal lungs were isolated and samples were prepared for paraffin sections. Sections were stained with the following primary rabbit antibodies: anti-MCRIP1 (Atlas; 1:400); anti-SP-B (Santa Cruz; 1:300); anti-SP-C (Abcam; 1:1000), anti-CtBP1 (Abcam; 1:500) and anti-CtBP2 (Abcam; 1:500); anti-Foxp1 (CST; 1:200) and anti-Foxp2 (CST; 1:2000).

### RNA extraction and qRT-PCR analyses

Mouse neonates were killed according to animal experiment regulations and lung tissues were isolated. Isolated lung tissues were immediately treated with Trizol and disrupted using tissue homogenizers. For qRT-PCR analysis using the mouse AEC2 cell line, MLE12, the cells were pre-seeded 24 h prior to RNA extraction. Total RNA was extracted, precipitated by the ethanol precipitation method, and was then reverse-transcribed using the PrimeScript RT Master Mix (Perfect Real Time) (Takara). Reverse-transcribed complementary DNA was used for real-time PCR quantification. RT-PCR was performed using the Thunderbird SYBR qPCR Mix (TOYOBO) and the following primers: *Gapdh* forward, 5′-CCGCATCTTCTTGTGCAGTG-3′; *Gapdh* reverse, 5′-GATGGGCTTCCCGTTGATGA-3′; *Sftpb* forward, 5′-CCAAGTGCTTGATGTCTACC-3′; *Sftpb* reverse, 5′-CTGGATTCTGTTCTGGCTTA-3′; *Sftpc* forward, 5′-GTAGCAAAGAGGTCCTGATG-3′; *Sftpc* reverse, 5′-CCTACAATCACCACGACAA-3′; *Muc1* forward, 5′-GTCTTCAGGAGCTCTGGTGG-3′; *Muc1* reverse, 5′-TACCACTCCAGTCCACAGCA-3′; *Abca3* forward, 5′-GCATTGCCCTCATTGGAGAGCCTG-3′; *Abca3* reverse, 5′-TCCGGCCATCCTCAGTGGTGGG-3′. Each target gene was amplified using a 2-step PCR program.

### siRNA knockdown experiment

A549 and MLE12 cells were transfected with siRNAs targeting human *MCRIP1* or mouse *Mcrip1* using Lipofectamine RNAiMAX (Invitrogen). The targeting sequence was as follows: si_hMCRIP1#1, 5′-CAGAGUCGUGUACAACGGCAAdTdT-3′; si_mMCRIP1#1, 5′-GACCUCUGUUUCUUCGGCUdTdT-3′; si_mMCRIP1#2, 5′-GUGCCUAGUGGAGGAGUAUdTdT-3′.

### Dual luciferase reporter assay

Cells were seeded in a 24-well plate and transfected with the luciferase reporter plasmids, pGL4-SP-B (−3000/+450) or pGL4-SP-C (−3000/+120), pcDNA3-Foxp2, pcDNA3-CtBP2, and pcDNA3-MCRIP1 as indicated, together with the Renilla luciferase plasmid as an internal control. The cells were assayed for luciferase activity 48 h after transfection, using Twinlite Firefly and Renilla Luciferase Reporter Gene Assay System (Perkin Elmer).

### ChIP assay

The ChIP assays were performed using the SimpleChIP Enzymatic Chromatin IP Kit (CST) according to the manufacturer’s protocol. Immunoprecipitants were recovered overnight with specific antibodies or with normal rabbit IgG (CST). The purified DNA was amplified by PCR using the SP-B promoter-specific primers: *Sftpb* forward, 5′-CAGACAGAAGTCATCCTTGTTGAATG-3′; *Sftpb* reverse, 5′-ACATGGTACCGACTTGGCC-3′. PCR was performed under the following conditions: 95 °C for 5 min, followed by 30–35 cycles of 95 °C for 30 sec, 58 °C for 30 sec and 72 °C for 30 sec, and a final step of 72 °C for 5 min. The PCR products were subjected to 2% agarose gel electrophoresis. The following rabbit antibodies were used: anti-Histone H3 (CST; 1 μg/sample), anti-Pan-Methyl-Histone H3 (K9) (CST; 1 μg/sample), anti-Acetyl-Histone H3 (K9) (CST; 1 μg/sample), anti-CtBP1 (Abcam; 1 μg/sample), anti-Foxp1 (CST; 1 μg/sample), and anti-Foxp2 (Abcam; 1 μg/sample).

### Establishment of MCRIP1-KO cell lines

To construct CRISPR/Cas9 plasmids targeting mouse *Mcrip1*, target genomic DNA sequences were designed using the online design tool, CRISPRdirect (https://crispr.dbcls.jp). The following oligos were then designed according to each specific target genomic DNA sequence: m*Mcrip1* KO#1 forward, 5′-CACCGTCTCCAGAGTTGTCTACAA-3′; m*Mcrip1* KO#1 reverse, 5′-AAACTTGTAGACAACTCTGGAGAC-3′; m*Mcrip1* KO#2 forward, 5′-CACCGAGTTGTCTACAACGGCAAG-3′; m*Mcrip1* KO#2 reverse, 5′-AAACCTTGCCGTTGTAGACAACTC-3′. The sgRNA was prepared and inserted into the pSpCas9-Puro expression plasmid. MLE12 cells were transfected with a pSpCas9-puro expression vector carrying m*Mcrip1* KO#1 or KO#2 gRNA using X-treme Gene 9 (Sigma). Puromycin-selected cells were diluted and sub-cloned into 96-well plates in order to collect single-cell clones.

### Statistics and reproducibility

Data represent means ± SEM of at least three independent experiments. The statistical significance of the difference between mean values was tested using Student’s *t* test, except for the distribution analysis of *Mcrip1* progeny, which was analyzed using a *χ*^2^ test.

### Reporting summary

Further information on research design is available in the Nature Research Reporting Summary linked to this article.

## Supplementary information

Supplementary Information

Description of Additional Supplementary Files

Supplementary Data 1

Supplementary Movie 1

Reporting Summary

## Data Availability

All relevant data generated or analyzed during this study are included in this article and Supplementary Information. All other data are available from the authors upon reasonable request. The source data underlying the plots in figures are provided in Supplementary Data 1. Full scan images of the gel electrophoresis and western blot data are shown in Supplementary Fig. 6.
