# Supplementary material for: MCRIP1 promotes the expression of lung-surfactant proteins in mice by disrupting CtBP-mediated epigenetic gene silencing
Source: Commun Biol. 2019 Jun 20;2:227. doi: 10.1038/s42003-019-0478-3 (PMC6586819; doi:10.1038/s42003-019-0478-3)
Supplement: Supplementary file 1 — Supplementary Information [file 42003_2019_478_MOESM1_ESM.pdf]

a

Distribution of *Mcrip1* progeny by chi-square test

Genotype	Observed	value	Expected	Freq. Observed (%)	Freq. Expected (%)
WT (AA)	50	HIGH	39	32.05	25
Het (Aa)	88	HIGH	78	56.41	50
KO (aa)	18	LOW	39	11.54	25
Totals	156		156	100.00	100

Chi-square calculated value=	15.692	NON-MENDELIAN
Chi-square table=	5.991	
Confidence Level=	0.05	
Calculated p-value=	0.000391254	
Degress of Freedom=	2	

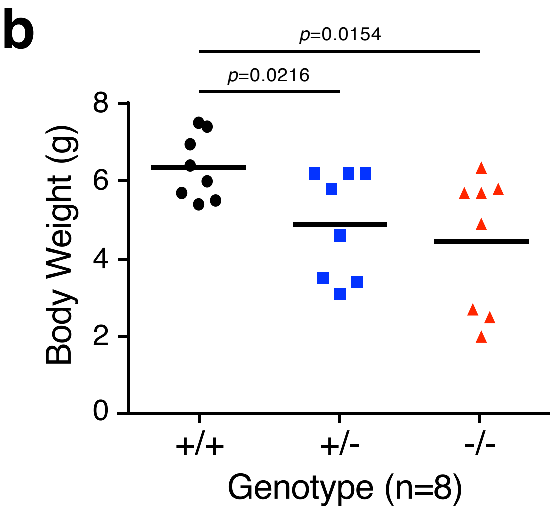

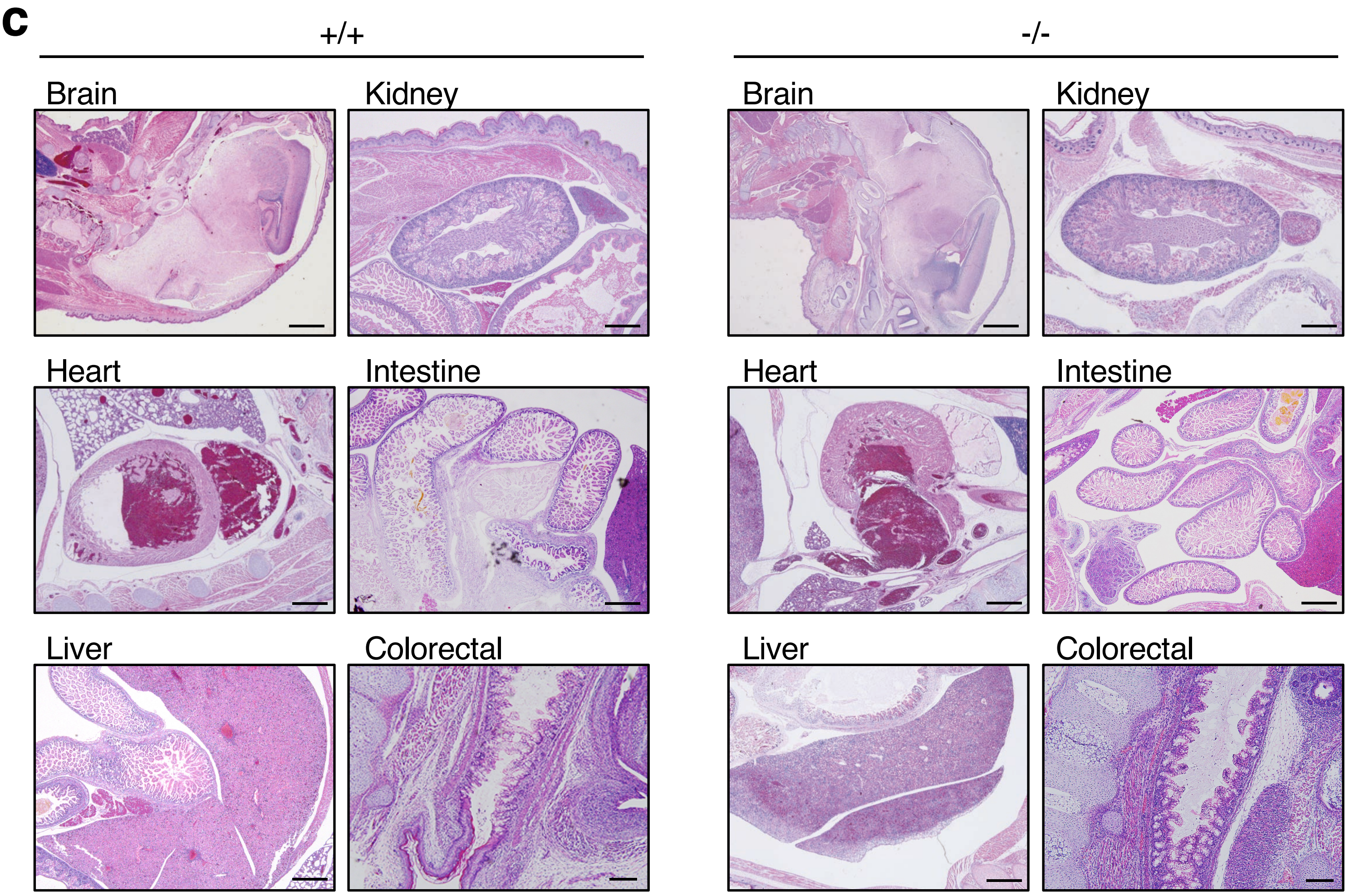

Supplementary Figure 1. *Mcrip1*-KO mice die at the neonatal stage due to respiratory failure.
(**a**) Analysis of *Mcrip1* progeny distribution by a chi-square test. Heterozygous (Het, Aa) mating was repeated to obtain homozygous mutant mice (KO, aa). A pool of 156 mice was recorded, as shown in Fig. 1d, and was used for analysis of the survival of *Mcrip1* progeny. "Observed" indicates the actual counts of each genotype (WT, AA; Het, Aa; KO, aa), and "Expected" indicates the calculation from the total pool based on Mendel's law. "Frequency (Freq.) Observed" was obtained by calculation of the proportion of the observed number in the total pool. A chi-square test was performed to obtain the p-value.
(**b**) Body weight analysis of mice that were still alive at the age of 8 weeks. +/+, wild-type; +/-, heterozygous; -/-, homozygous knockout mice.
(**c**) Gross histology of tissues of *Mcrip1*$^{+/+}$ and *Mcrip1*$^{-/-}$ mice analyzed by H&E staining. Scale bars, 100 μm.

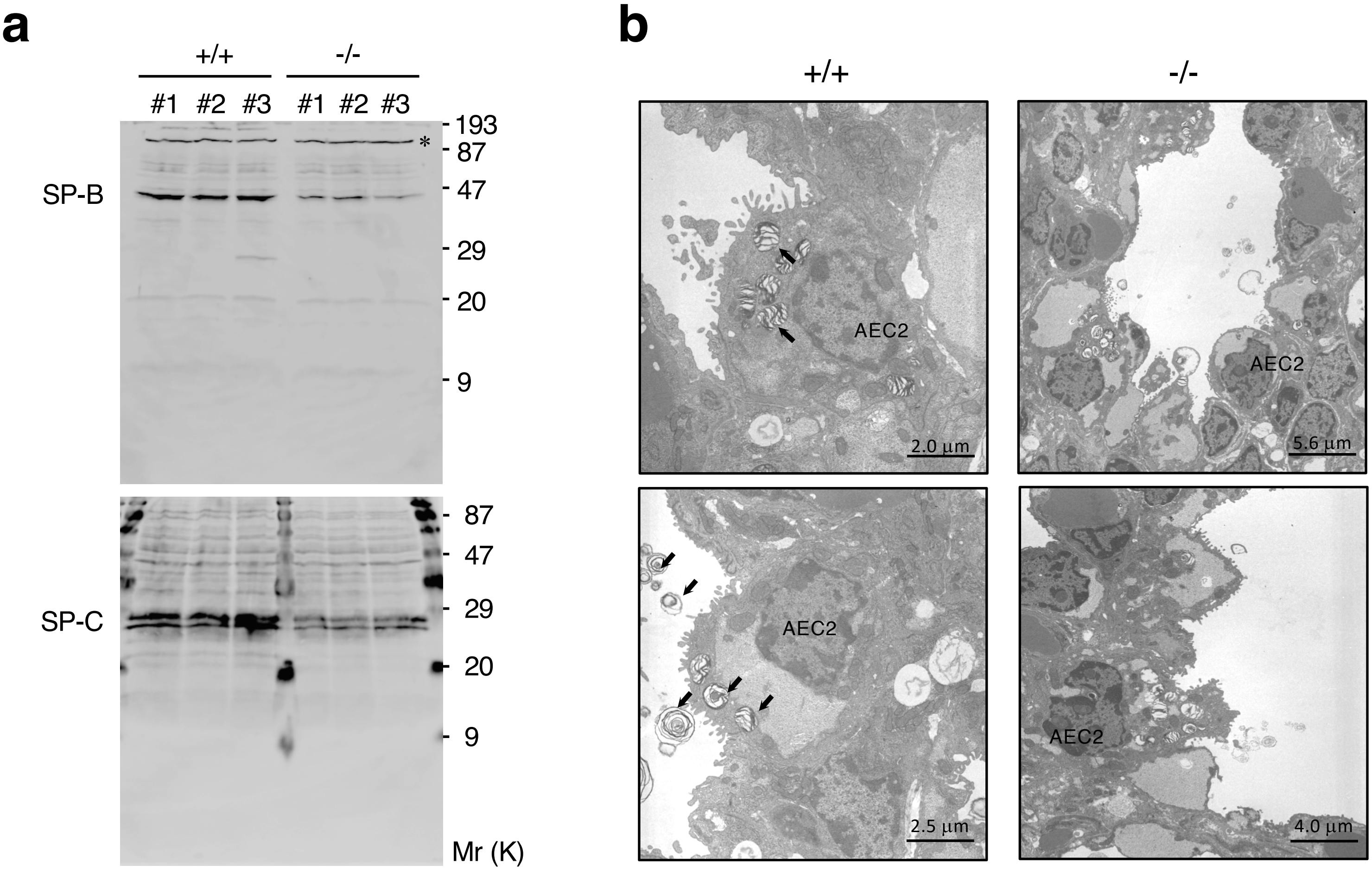

Supplementary Figure 2. Surfactnt proteins are down-regulated in Mcrip1-KO mouse lungs.

(**a**) Western blot analyses of SP-B and SP-C expression in whole lung lysates isolated from *Mcrip1*$^{+/+}$ and *Mcrip1*$^{-/-}$ mice. A non-specific background band of immunoblotting is shown as a loading control (asterisk).

(**b**) Ultrastructure of type 2 alveolar epithelial cells. Ultrastructure of type 2 alveolar epithelial cells (AEC2) and lamellar bodies in *Mcrip1*$^{+/+}$ and *Mcrip1*$^{-/-}$ lungs at embryonic day 21 (E21). Arrows indicate lamellar bodies. Scale bar lengths are indicated.

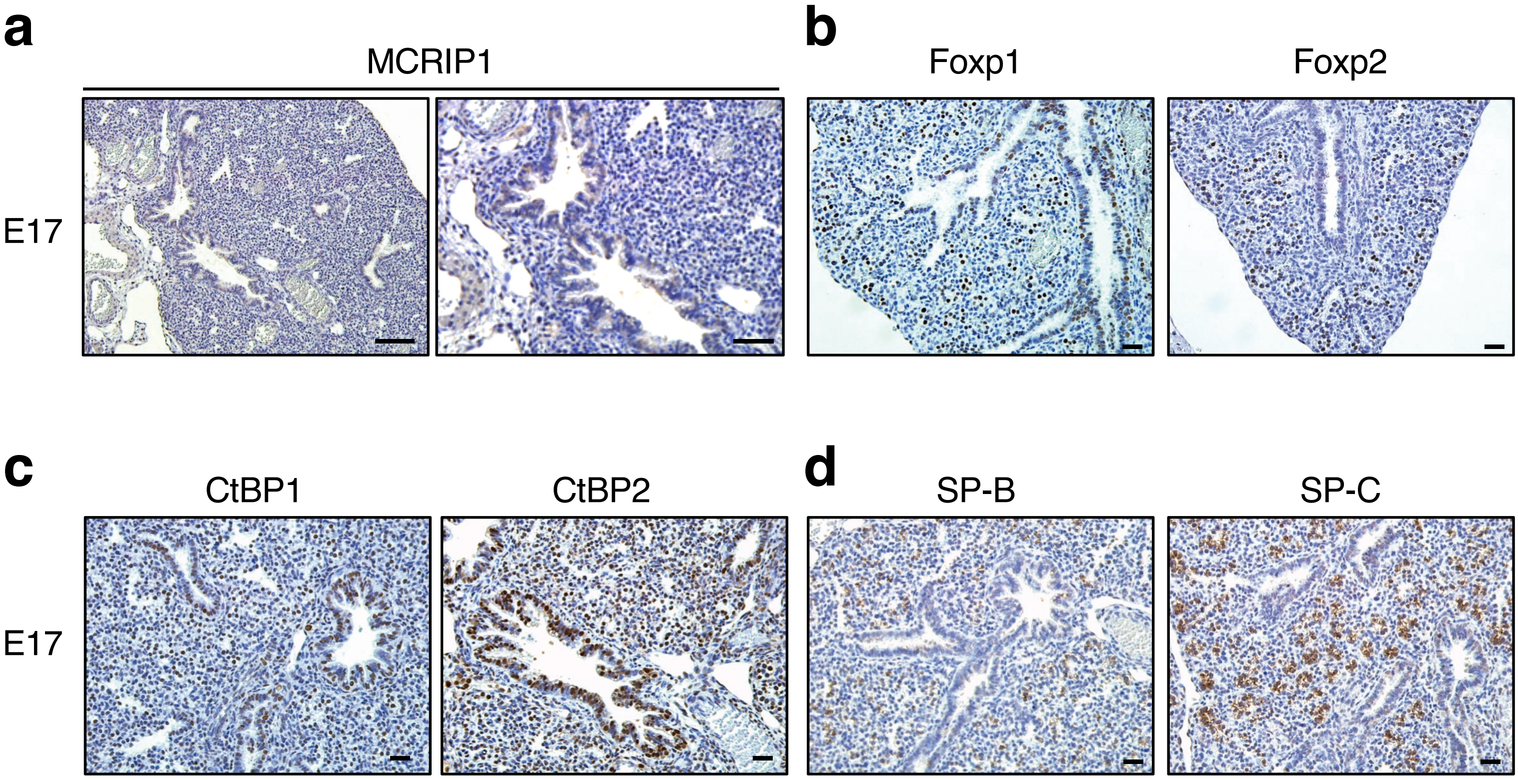

Supplementary Figure 3. MCRIP1 is expressed in the lung epithelium during lung development. (**a**-**d**) Immunohistochemical analysis of the expression of MCRIP1(**a**), Foxp1 and Foxp2 (**b**), CtBP1 and CtBP2 (**c**), and surfactant protein B (SP-B) and surfactant protein C (SP-C) (**d**) in *Mcrip1*$^{+/+}$ lungs at the embryonic stage of E17. Scale bars, 100 and 50 μm in left and right panels of (**a**), respectively. Scale bar, 50 μm in (**b**-**d**).

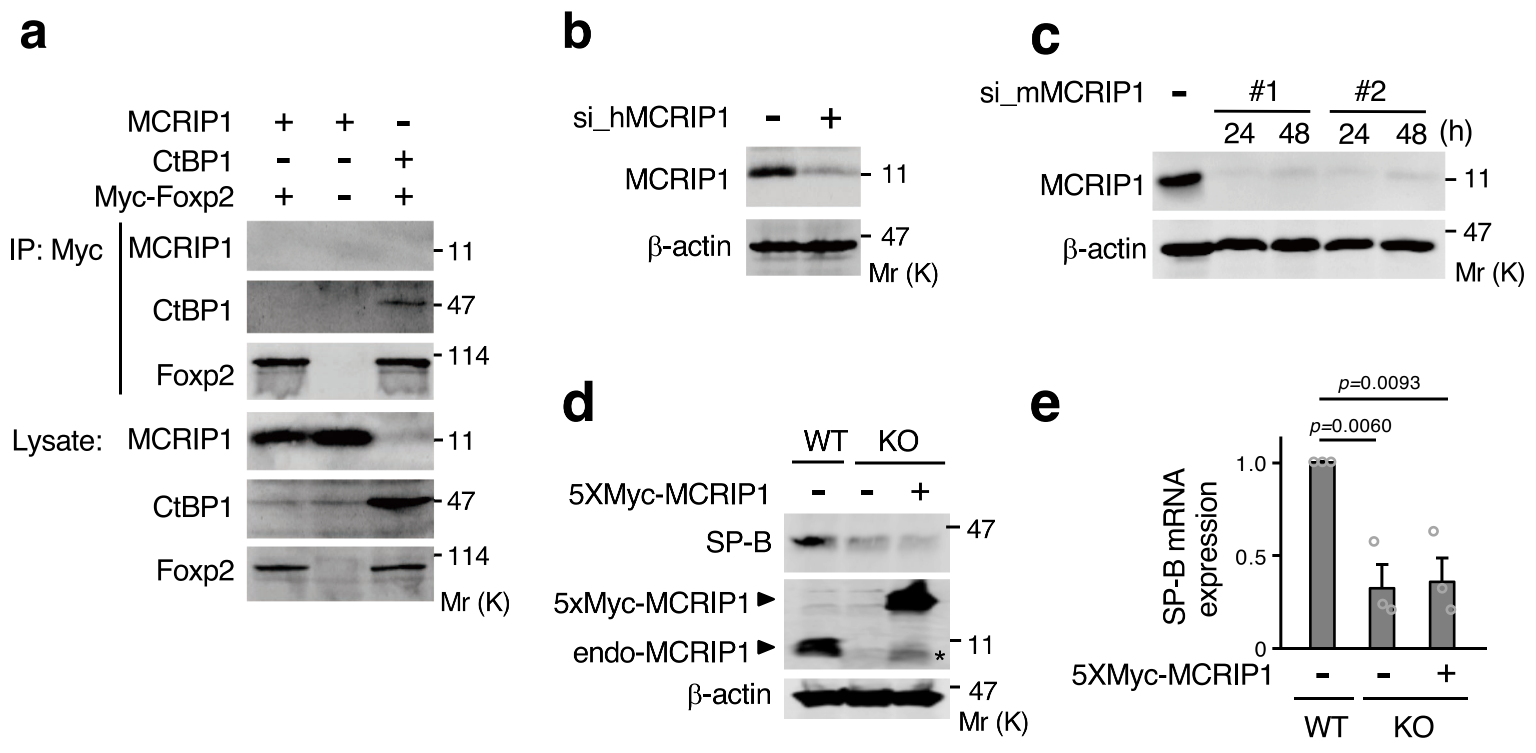

Supplementary Figure 4. MCRIP1 depletion allows the recruitment of a CtBP corepressor complex to the SP-B promoter and modulates histone modifications.

(**a**) MCRIP1 does not interact with Foxp2. HeLa cells were transiently transfected as indicated. Myc-Foxp2 was immunoprecipitated using an anti-Myc antibody, and coprecipitated MCRIP1 and CtBP1 were probed with anti-MCRIP1 and anti-CtBP1 antibodies, respectively. In contrast to CtBP1, MCRIP1 does not bind to Foxp2.

(**b**) Knockdown efficiency of siRNA targeting human MCRIP1. Human A549 cells were transfected with siRNA (si_hMCRIP1) and incubated for 48 hours. Whole-cell lysates were prepared and analyzed by immunoblotting with an anti-MCRIP1 antibody.

(**c**) Knockdown efficiency of siRNA targeting mouse MCRIP1. Mouse MLE12 cells were transfected with siRNAs (si_mMCRIP1#1 and #2) and incubated for 24 or 48 hrs. Whole-cell lysates were prepared at the indicated time points and were analyzed by immunoblotting.

(**d**, **e**) SP-B expression in wild-type (WT) or *Mcrip1*-KO MLE12 cells. Where indicated (+), 5xMyc-tagged MCRIP1 was re-introduced into *Mcrip1*-KO MLE12 cells. In (d), the expression level of endogenous SP-B proteins was analyzed by immunoblotting (top). The asterisk shows a degradation product of 5xMyc-MCRIP1. In (e), the expression levels of SP-B mRNA was analyzed using qRT-PCR. The data represent the mean±SEM from three independent experiments. Reintroduction of MCRIP1 did not restore SP-B expression in *Mcrip1*-KO MLE12 cells, because once MCRIP1 was genetically ablated in cells, the SP-B gene was epigenetically silenced by altering the pattern of histone modifications (see the main text for details). (**b**, **c, d**) β-actin, a loading control.

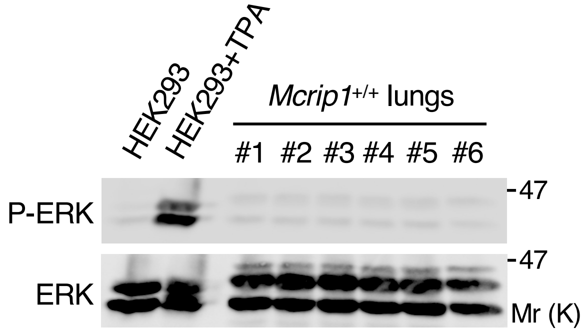

Supplementary Figure 5. ERK is not activated in wild-type (*Mcrip1*$^{+/+}$) lungs.

The lungs of *Mcrip1*$^{+/+}$ newborn mice (#1-6) were isolated and their extracts were probed for phosphorylated-ERK1/2 (P-ERK) by immunoblotting (top). The same filter was stripped and reprobed with an anti-ERK1/2 antibody (bottom) to ensure equal loading of proteins among the samples. As a positive control for ERK activation, HEK293 cells were treated with a phorbol ester, 12-O-tetra-decanoyl phorbol 13-acetate (TPA; 100 nM for 30 min).

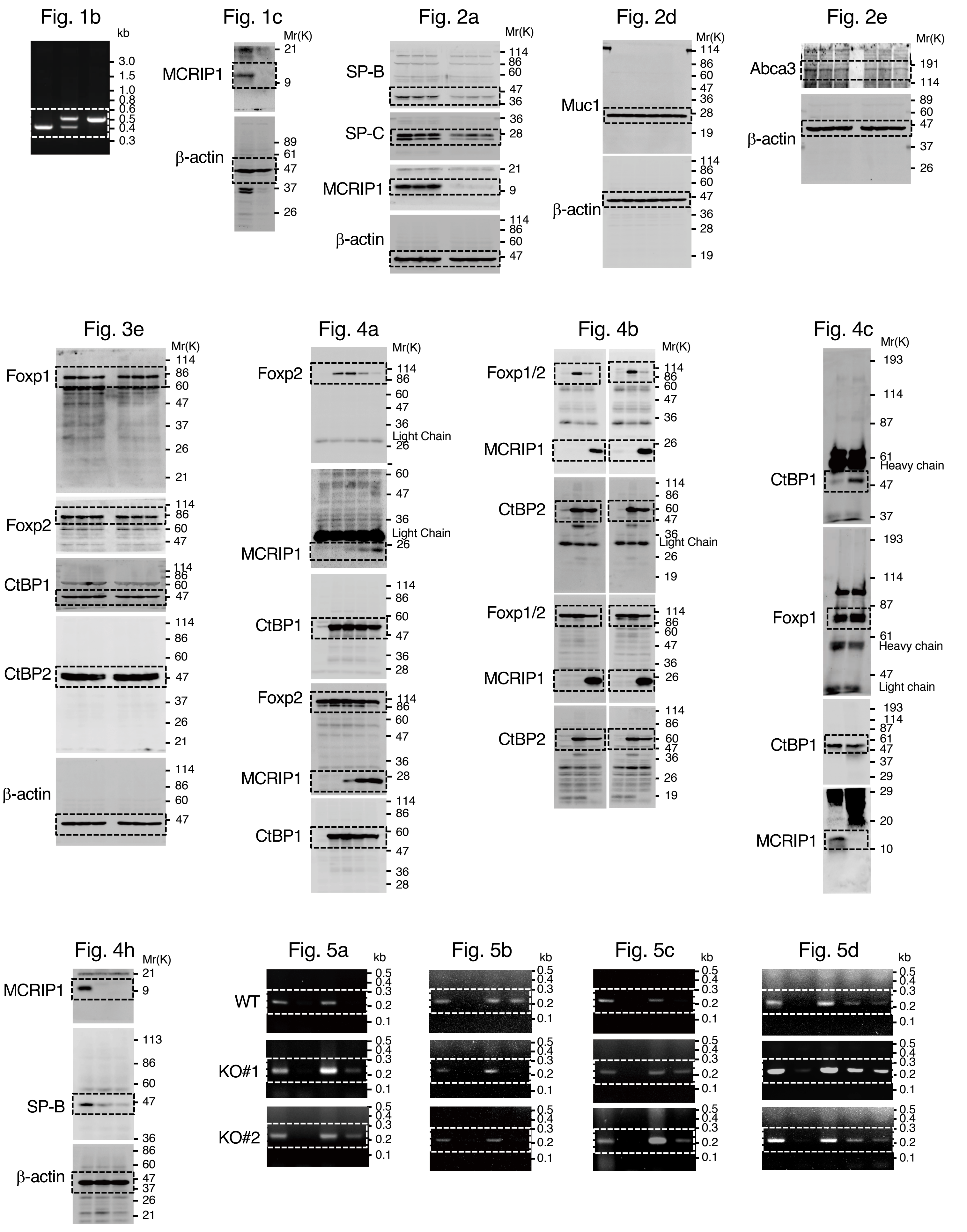

Supplementary Figure 6. Full scan images of gel/western data.

(continued)

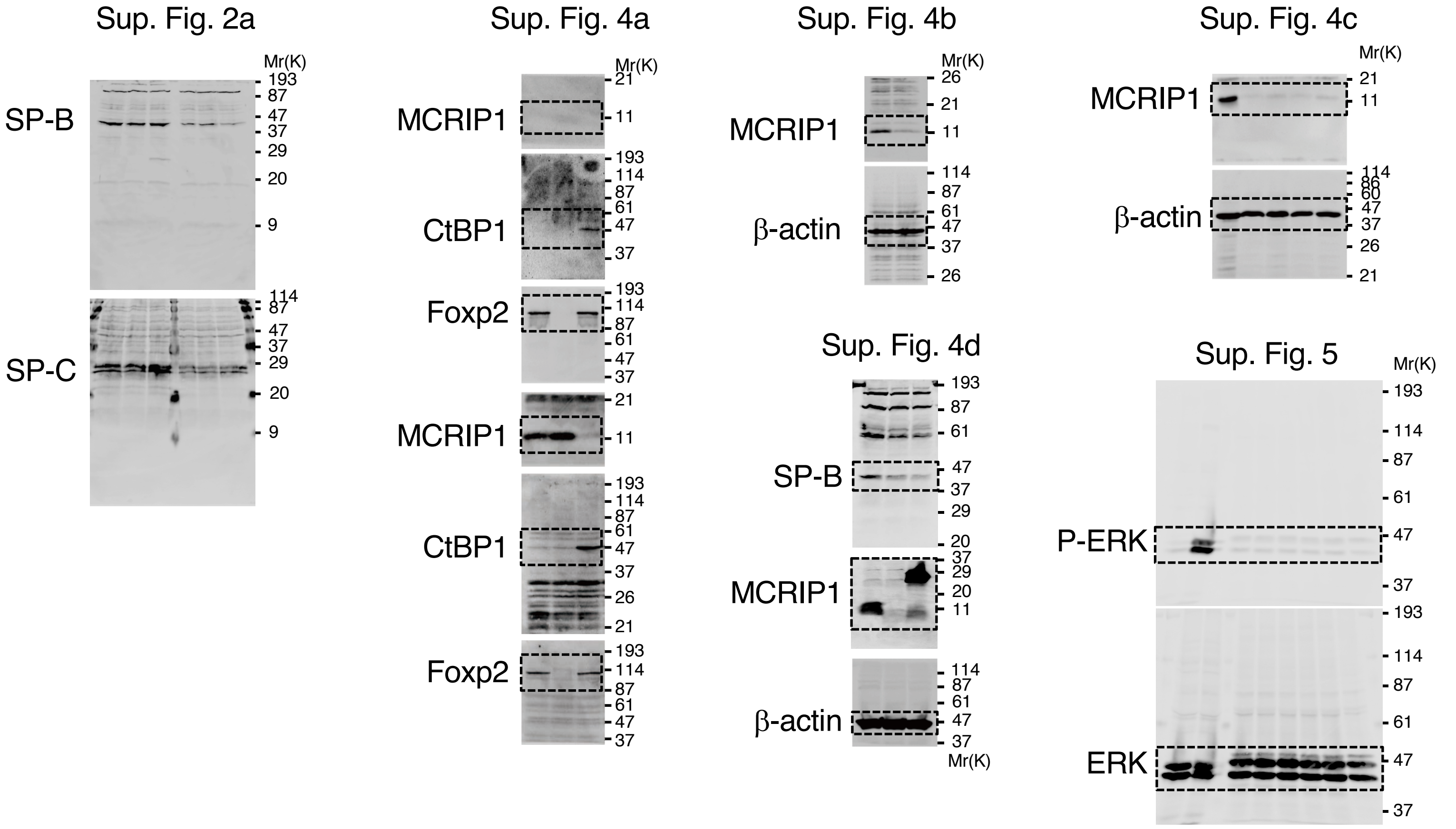

Supplementary Figure 6. Full scan images of gel/western data.
