# Supplementary material for: MCRIP1 promotes the expression of lung-surfactant proteins in mice by disrupting CtBP-mediated epigenetic gene silencing
Source: Commun Biol. 2019 Jun 20;2:227. doi: 10.1038/s42003-019-0478-3 (PMC6586819; doi:10.1038/s42003-019-0478-3)
Supplement: Supplementary file 2 — Description of Additional Supplementary Files [file 42003_2019_478_MOESM2_ESM.docx]

**Description of Additional Supplementary Files**

**File Name**: Supplementary Data 1

**Description**: The source data underlying the plots in figures.

**File Name**: Supplementary Movie 1

**Description**: Phenotype of *Mcrip1^-/-^* mice. *Mcrip1^-^*^/-^ newborn pups exhibited gasping breath, tachypnea and cyanosis.
